# Patient Education Using AI: A Cross-Sectional Study Comparing ChatGPT and Google Gemini-Generated Patient Education Brochures on Various Surgical Management of Breast Cancer

**DOI:** 10.7759/cureus.97525

**Published:** 2025-11-22

**Authors:** Mohamed Naufal, Sudharshan Kannan, Swetha Senthilkumar, Tilagaraj Palaniappan, Sooraj S Kumar, Pallavi Padmakar Kulkarni

**Affiliations:** 1 Plastic Surgery, Mahatma Gandhi Medical College and Research Institute, Pondicherry, IND; 2 General Surgery, Government Medical College Thiruvannamalai, Thiruvannamalai, IND; 3 Psychiatry, Shaditya Hospital, Chennai, IND; 4 General Surgery, PSG Institute of Medical Sciences, Coimbatore, IND; 5 General Surgery, Plastic Surgery, Grant Medical College Mumbai, JJ Hospital Mumbai, Mumbai, IND

**Keywords:** artificial intelligence, chatgpt, education tools, google gemini, modified radical mastectomies

## Abstract

Introduction

Artificial intelligence tools such as ChatGPT and Google Gemini are increasingly used for generating patient education materials. This study aimed to compare the readability, reliability, and originality of artificial intelligence (AI)-generated patient education content on surgical management options for breast cancer.

Methods

A cross-sectional study was conducted from January 15 to 21, 2025. Standardized prompts requesting patient education brochures for common breast cancer surgeries were given to ChatGPT-4o and Gemini 2.0 Flash. All responses were generated in English. Readability was assessed using the validated Flesch-Kincaid Readability Calculator, originality using a similarity checker, and reliability using the Modified DISCERN tool. Statistical comparisons were performed using unpaired t-tests with significance set at p<0.05.

Results

There were no significant differences between the two AI tools in syllables per word, ease score, similarity, or reliability. ChatGPT produced significantly higher word counts (p=0.018) and sentence counts (p=0.001). Gemini generated longer sentences and a higher grade-level score (p=0.002), indicating relatively more complex text.

Conclusions

Both AI tools produced patient education materials with broadly comparable readability, reliability, and originality. No correlation was observed between readability ease and reliability scores, suggesting consistent performance across both platforms.

## Introduction

Breast cancer is the most frequently diagnosed cancer among women worldwide, representing 11.6% of all new cases. [1] Over the past several decades, medical decision-making has shifted from a paternalistic model - where physicians determined the best treatment based on their own judgment of the patient’s best interest - to one centered on patient autonomy, allowing informed patients to make their own treatment choices based on their values and priorities. In modern medical ethics, shared decision-making is considered the ideal approach, balancing patient autonomy with the physician’s expertise. This process ensures patients are informed about the most effective treatment options, incorporates their values and preferences, and enhances communication between patients and clinicians [1].

Patients newly diagnosed with curable breast cancer must navigate a complex treatment decision-making process. This challenge has driven efforts to enhance decision quality, ensuring that patients are well-informed about the risks and benefits of various treatments, that their values are recognized and integrated into treatment choices, and that they feel prepared to actively participate in discussions with their clinicians [2]. Decision aids have been developed to assist patients in navigating treatment choices following a breast cancer diagnosis [3]. Only a few trials have assessed the use of decision tools for breast cancer treatment in surgical practice [4]. Only one study has shown that a decision aid positively impacts key aspects of decision quality, such as knowledge and decision satisfaction [5].

In contrast, other studies have yielded mixed results. The effectiveness of existing tools is constrained by small patient samples drawn from a single academic center or a limited number of clinical sites, as well as by insufficient consideration of the diverse decision-making workflows in community practice. While numerous cancer treatment websites and some online decision aids are available, significant gaps remain in patients' understanding of their treatment options, even after undergoing surgery [6]. Additionally, patients express a strong need for support in making cancer treatment decisions [7]. Breast cancer patients would demonstrate higher rates of high-quality decision-making, defined as being both well-informed and aligned with their values, along with greater readiness to make treatment decisions and a more positive appraisal of their choices, compared to those who viewed the control version, which resembled standard contemporary websites [8].

The aim of this study is to assess the readability and comprehensibility of responses generated by ChatGPT and Google Gemini for the composition of a patient education guide on surgical treatments for breast cancer, a comparative analysis.

## Materials and methods

This cross-sectional original research study was conducted over a one-week period, from January 15 to January 21, 2025. The primary aim was to assess the suitability of artificial intelligence (AI)-generated content for patient education regarding the surgical management of breast cancer. As the study did not involve human participants, ethics committee approval was not required.

The study focused on surgical procedures for breast cancer, and two AI models were selected for generating patient education material: ChatGPT-4o (OpenAI, accessed on January 19, 2025) and Gemini 2.0 Flash (Google DeepMind, accessed on January 19, 2025). To ensure consistency, standardized prompts were developed and provided to both models. Each prompt requested the generation of a patient education guide on specific breast cancer surgeries, including simple mastectomy, radical mastectomy, modified radical mastectomy, partial mastectomy, breast-conserving surgery (lumpectomy), and nipple-sparing mastectomy. The responses generated by each model were exported directly into Microsoft Word without modification and compiled for evaluation.

The responses were assessed across three main domains. First, readability was analyzed using the Flesch-Kincaid Readability Calculator, which provided measures of word count, sentence count, ease of understanding, and grade-level equivalence. Second, originality of the content was evaluated using the QuillBot Plagiarism Tool (version 18.3.0), which compared the AI outputs against existing online sources to detect similarity. Third, the reliability of the generated patient education materials was assessed using the Modified DISCERN instrument, a validated tool designed to evaluate the quality and trustworthiness of health information [9-11].

All data were initially organized in Microsoft Excel and subsequently analyzed using R software, version 4.3.2 (R Core Team, 2023). Mean scores from ChatGPT-4o and Gemini 2.0 Flash were compared using an unpaired t-test, with a p-value less than 0.05 considered statistically significant. To further explore associations between readability and reliability, correlation analysis was performed using Pearson’s correlation coefficient, assessing the relationship between the Flesch-Kincaid readability scores and Modified DISCERN scores. To enhance study quality and reduce bias, identical prompts were consistently applied to both AI models, independent validated tools were used for evaluation, and data analyses were performed independently by the authors to ensure transparency and reproducibility.

Tool accessibility and licensing: All assessment tools used in this study were freely available or appropriately licensed for academic use. The Flesch-Kincaid Readability Calculator and the Modified DISCERN instrument are publicly accessible and free to use for non-commercial research. Statistical analysis was performed using R software (version 4.3.2, R Foundation for Statistical Computing, Vienna, Austria), which is open-source under the GNU General Public License. The QuillBot Plagiarism Checker (version 18.3.0, QuillBot Inc., Chicago, IL, USA) was accessed through a licensed account to verify the originality of content.

## Results

ChatGPT and Google Gemini were used to generate patient education brochures for six commonly performed breast cancer surgeries: simple mastectomy, radical mastectomy, modified radical mastectomy, partial mastectomy, breast-conserving surgery (lumpectomy), and nipple-sparing mastectomy. The outputs from both AI models were analyzed for readability, similarity, and reliability.

Table 1 summarizes the characteristics of responses generated by the two models. ChatGPT produced responses with a significantly higher word count (mean 584.17 ± 79.48) compared to Google Gemini (mean 481.50 ± 40.20, p = 0.018). Similarly, the mean sentence count in ChatGPT outputs (84.50 ± 17.66) was almost double that of Gemini (46.50 ± 8.04), and this difference was highly significant (p = 0.001). These findings suggest that ChatGPT tends to generate more elaborate and detailed brochures.

**Table 1:** Characteristics of responses generated by ChatGPT and Google Gemini. The statistical test used is the t-test. P-values <0.05 are considered statistically significant.

Parameter	ChatGPT (Mean ± SD)	Google Gemini (Mean ± SD)	t-value	p-value
Words	584.17 ± 79.48	481.50 ± 40.20	2.91	0.018*
Sentences	84.50 ± 17.66	46.50 ± 8.04	5.13	0.001*
Average Words per Sentence	7.08 ± 1.23	10.53 ± 1.40	-5.29	0.001*
Average Syllables per Word	1.85 ± 0.06	1.88 ± 0.10	-0.73	0.489
Grade Level	8.98 ± 0.63	10.75 ± 0.85	-4.27	0.002*
Ease Score	43.15 ± 4.26	36.82 ± 7.36	1.85	0.098
Similarity (%)	37.83 ± 19.40	33.88 ± 16.69	0.39	0.713
Reliability Score	3.67 ± 0.52	4.00 ± 0.00	-1.49	0.175

However, when sentence complexity was examined, Google Gemini generated longer sentences, with an average of 10.53 ± 1.40 words per sentence compared to 7.08 ± 1.23 words in ChatGPT outputs (p = 0.001). This structural difference was also reflected in the readability grade level: ChatGPT brochures corresponded to a lower mean grade level of 8.98 ± 0.63, whereas Gemini brochures were written at a higher grade level of 10.75 ± 0.85 (p = 0.002). This indicates that Gemini’s responses required a higher literacy level for comprehension, while ChatGPT produced relatively easier-to-read material.

No significant differences were observed in the average syllables per word (p = 0.489), suggesting comparable linguistic complexity in terms of vocabulary across both models. Similarly, the Flesch-Kincaid ease of reading score showed no significant difference (p = 0.098), although ChatGPT demonstrated a trend toward greater readability (43.15 ± 4.26 vs. 36.82 ± 7.36).

Analysis of originality revealed that the mean similarity percentage was higher in ChatGPT (37.83 ± 19.40) than in Gemini (33.88 ± 16.69), but this difference was not statistically significant (p = 0.713). Both models thus showed comparable performance in generating non-plagiarized content.

Finally, the reliability of educational content, assessed using the modified DISCERN score, demonstrated no statistically significant difference between the two models (p = 0.175). While Gemini consistently achieved a reliability score of 4.00, ChatGPT had a slightly lower mean score of 3.67 ± 0.52.

## Discussion

A cross-sectional study comparing patient education brochures on various breast surgeries generated by ChatGPT and Google Gemini found that ChatGPT responses had significantly higher word and sentence counts, but lower average words per sentence and grade-level scores. Natural language processing (NLP) is a fundamental component of AI, encompassing the methods by which humans interact using language. Large language models (LLMs), driven by deep learning algorithms, perform diverse NLP tasks including question answering, language translation, and document summarization [12].

In a study that compared physician-written and AI-generated replies to patients’ queries, AI-generated replies were rated to be more empathetic than physician-written responses [13]. Hence, AI chatbots may provide an irreplaceable source of patient education in the future, particularly if prompted to provide results with better readability [14].

Readability of chatbot responses is crucial for their effective use, as patient comprehension of the language and terminology is essential. For patients to trust information provided by AI chatbots, the explanations must be easily understood. [15] Generally, Short, concise texts improve readability and retention. Using 8-10 words per sentence is recommended to enhance reading ease. [15] In this study, the average response generated by both the AI tools remains at the desired level of words per sentence.

For patient education brochures, the readability score should ideally correspond to a level that is easily understood by individuals with a high school education. It was calculated by using the Flesch-Kincaid Calculator, and the average ease score for ChatGPT is 43.15, and 36.82 for Gemini. Both of them are at the college level, which is difficult to understand. Comparing both, ChatGPT was slightly better to read. However, there was a statistically significant difference in Flesch-Kincaid grade level, in which Gemini performed better. In a 2024 study, Ghanem et al. assessed the readability of various AI platforms using the Flesch Reading Ease Score (FRES) and the Flesch-Kincaid Grade Level (FKGL). Their study found that while all platforms generated responses that were difficult to read, Google Bard demonstrated the highest readability among them. [16] Plagiarism in AI chatbots is a growing concern, particularly when they generate responses resembling existing copyrighted content. As AI is trained on large datasets, it invariably includes publicly available texts, which may contain copyrighted material. Another significant problem is that most AI models do not provide citations unless specifically designed to do so. Plagiarism in medical science is more than just academic dishonesty-it can have real-world consequences affecting patient care, scientific credibility, and the overall integrity of the medical field. Proper citation, ethical research practices, and originality are essential to maintaining trust and progress in medicine.

The modified DISCERN score is a tool used to assess the quality and reliability of health information, particularly online patient education materials. It is a variation of the original DISCERN instrument, which was designed to evaluate consumer health information on treatment choices. Components of the modified DISCERN score are comprehensibility, reliability, balanced presentation, coverage of the topic, and transparency of sources. The Average DISCERN score of this study is 3.67 for ChatGPT and 4.0 for Gemini, and there is no statistical significance. In a recent study, Researchers evaluated responses from four AI chatbots (ChatGPT, ChatSonic, Perplexity, and Microsoft Bing AI) to the five most frequent Google Trends search queries related to urological cancers. The chatbot responses demonstrated moderate to excellent informational accuracy, with a median DISCERN score of 4 out of 5 (range 2-5), and contained no false information. [10, 17]

A study assessing the reliability and readability of ChatGPT-generated information on shoulder stabilization surgery found that responses were generally of good quality. However, the researchers highlighted that the reading level was challenging and noted the absence of cited source material. [18] Pan et al. evaluated responses from four AI chatbots (ChatGPT-3.5, Perplexity, ChatSonic, and Bing AI) to the five most common cancer-related search queries. They found the responses to be of high quality but written at a college reading level based on the FKGL score. The authors concluded that these tools should serve as supplementary rather than primary sources of medical information. [19]

Limitations

This original research analyzes two of the recent popular chatbots in India, whereas analysis of other AI chatbots is yet to be done by more original research. Likewise, the authors of this study have conducted this original research regarding the patient education manual provided by AI chatbots for only a limited amount of data; future studies are yet to be conducted on various other diseases, courses, and treatment methods. As this research was done with a small dataset, no major statistical significance was found between the two AI bots, but upcoming studies need to explore a similar study with a larger dataset so that any statistical significance may be obtained. Thus, AI tools may fail to adapt to rapidly evolving medical knowledge without human oversight, requiring continuous validation and expert supervision for patient education and clinical decision support.

## Conclusions

The study showed no significant differences in the average readability score, appropriateness grade, or reliability score between responses generated by the two AI tools for patient education brochures on the surgical treatment of breast cancer. Furthermore, there was no correlation between the ease score and reliability score of the two software programs.

To enhance the effectiveness of these tools, further research should be conducted to explore a wider range of AI tools and more recent diseases. Additionally, it is essential to assess whether these tools can generate content based on the latest guidelines. Furthermore, the tools should be enhanced to provide verifiable and accessible information to the general public.
